# Differential Diagnosis of Cavitary Lung Lesions

**DOI:** 10.5334/jbr-btr.1202

**Published:** 2016-11-19

**Authors:** Anagha P. Parkar, Panchakulasingam Kandiah

**Affiliations:** 1 Haraldsplass Deaconess Hospital, NO; 2 Haukeland University Hospital, NO

**Keywords:** Cavitary lung lesion, CT, Pulmonary infection, Pulmonary malignancy

## Abstract

Many different diseases present as cavitary pulmonary nodules. The spectrum of diseases ranges from acute to chronic infections, chronic systemic diseases, and malignancies. To decide on the most likely or correct diagnosis may be challenging. Knowledge of common and uncommon radiological findings in correlation with relevant clinical history and findings is necessary to make the right diagnosis and recommend the correct follow-up or step forward. The aim of this pictorial review is to present a brief overview of CT findings of common cavitary lung diseases seen in adult patients.

A cavity is defined in the Fleischner glossary as “a gas-filled space, seen as a lucency or low-attenuation area, within pulmonary consolidation, a mass, or a nodule” [1]. The cavity wall thickness may vary considerably. At their end-stage presentation, some cavitary diseases may present thin-walled cavities, or cysts. One should remember that there is a continuous transition from cavities to cysts. Cysts in the lungs are defined as “any round circumscribed space that is surrounded by an epithelial or fibrous wall of variable thickness”. The glossary further defines them as usually thin walled (i.e. < 2 mm) [1]. The wall thickness of the cavitary lung lesions in solitary disease can be useful in differentiating between benign and malignant disorders. A recent study found that a wall thickness of less than 7 mm was highly specific for benign disease, and a thickness of greater than 24 mm was highly specific for malignant disease. However, these thresholds are not absolute, as thin-walled carcinomas are also reported [2]. An additional indicator for malignancy was the lack of perilesional centrilobular nodules, whereas perilesional consolidation was common around benign nodules [3].

The content of the cavities is of little help in differentiating benign and malignant lesions. A benign bronchogenic cyst may contain fluid levels, as may a bronchoalveolar carcinoma. The degree of contrast enhancement in the content of nodules (< 10 HU) is shown to indicate benign lesions and may be used to distinguish aspergillomas from lung cancer [4,5]. Rim enhancement of the walls on contrast-enhanced CT is common in abscesses [6]. A connecting pulmonary artery may be seen in smaller metastases but not in larger ones, as the larger nodules tend to compress the vessels, so a lack of a feeding artery cannot be used to imply benign nodules [7].

The acute onset of symptoms is sometimes helpful to distinguish malignant and nonmalignant disease, but a benign infection may, for instance, cause hemoptysis when affecting a nearby vessel. Benign diseases may also cause fatigue and weight loss similar to malignancies. Acute onset of fever is usually helpful to distinguish benign disorders from malignancies, but a pulmonary cancer may present with a superinfection secondary to the tumor [8]. However, the combination of symptoms, laboratory results, past clinical history, and imaging findings leads to recognition of the correct diagnosis. This review presents the most commonly encountered cavitary lung diseases in adults in Europe.

## Infections

Several groups of microorganisms may cause cavitary lesions: common bacteria (for example, *Streptococcus p., Staph.aureus, Klebsiella p., H. influenzae*); typical and atypical mycobacterium; fungi (for example, aspergillosis, pneumocystis j.); and parasites [9].

### Pulmonary Abscess

**Figure 1:**
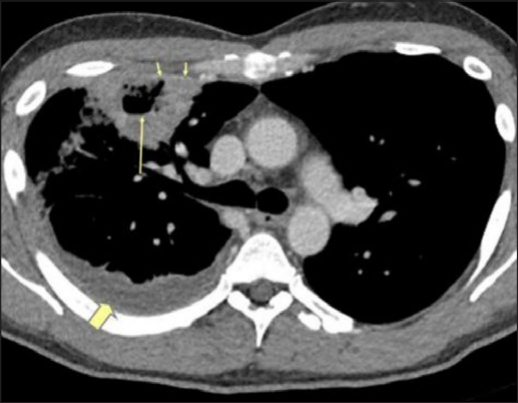
*Pulmonary abscess*. A 36-year-old male was admitted with sepsis a week earlier. A CT was performed due to low response to antibiotic treatment. A large cavity is seen in the right upper lobe with an air-fluid level (long arrow). Rim enhancement is seen in the cavity wall anteriorly (short arrows). A pleural effusion is also seen (thick arrow).

**Figure 2:**
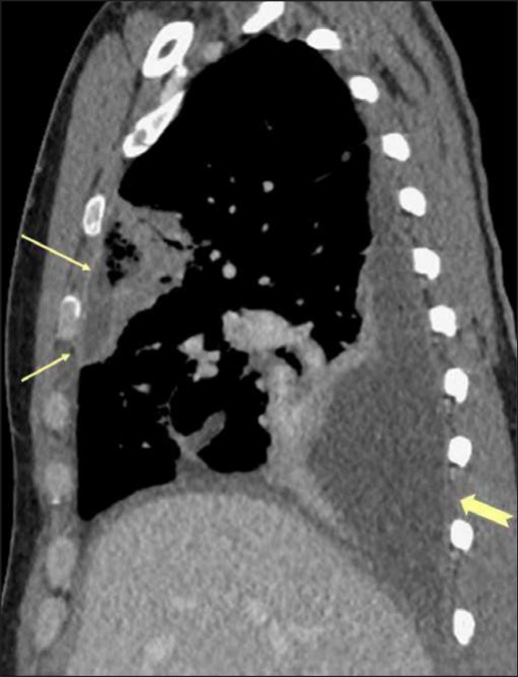
*Pulmonary abscess.* Same patient. The peripheral location has caused a local empyema (arrows), and there is also an enhancement of the pleura posteriorly, development of empyema (thick arrow).

Pulmonary abscess occurs as a complication of pneumonia. Symptoms are consistent with pneumonia: productive cough, fever, chest pain [10]. Most abscesses can be treated conservatively, but percutaneous drainage may be necessary in up to 20 per cent of cases [10]. In addition to such common complications as pneumothorax and empyema, the development of broncho-pleural fistulas is a rare complication [11]. Abscesses are seen on CT as cavitary lesions with or without a fluid level. They may occur anywhere in the lungs. Usually, intermediate to thick wall thickness with a peripheral contrast enhancement and necrotizing centre is visible. If the abscess is located peripherally, there may be local pleural thickening or an empyema (Figures 1, 2).

### Septic Emboli

**Figure 3:**
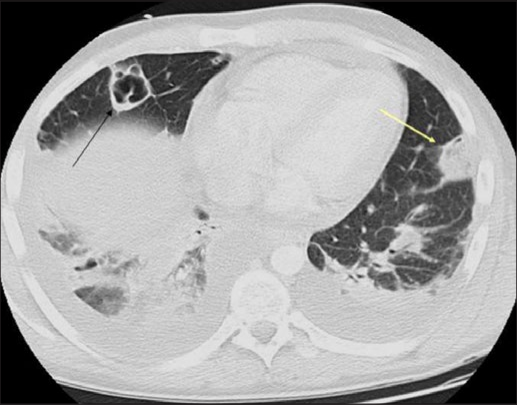
*Septic emboli*. A 29-year-old male and IV drug abuser was admitted with sepsis and dyspnea. There was a peripheral cavitary lesion in the right lung (black arrow) and a wedge-shaped lesion in the left lung (yellow arrow).

**Figures 4–6:**
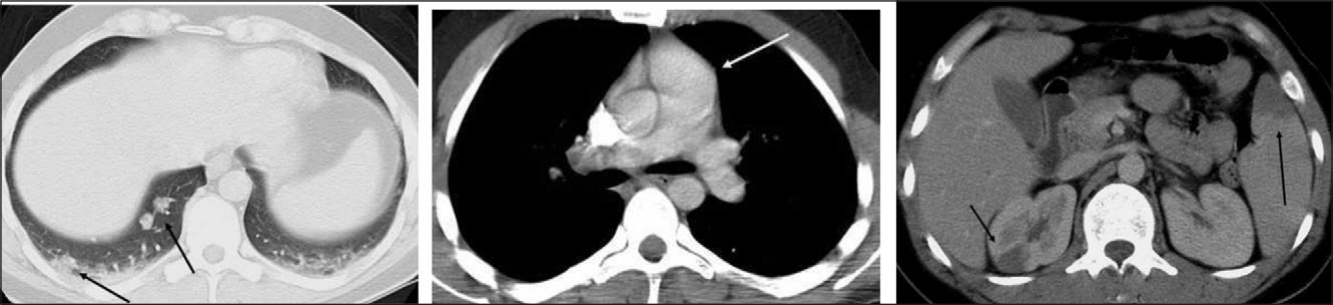
*Septic emboli* in the lungs and parenchymal organs. A 22-year-old male and IV drug abuser was admitted with chest pain. Peripheral nodules are seen, with an enlarged pulmonary artery, as well as infarctions in the spleen and right kidney (arrows) due to aortic endocarditis.

Septic emboli occur when microorganisms cause thrombosis in the peripheral pulmonary capillaries. Immunosuppressed patients, patients with arterial or intravenous catheters, intravenous drug abusers, and alcoholics and patients with endocarditis or those undergoing dental surgery are susceptible to septic emboli. The thrombi lead to infarction and consequent micro-abscesses. The patients are septic and have cough and dyspnea, chest pain, and perhaps hemoptysis and sinus tachycardia [12]. The CT presentation consists of multiple peripheral nodular or wedge-shaped opacities with a broad base against the pleura. The nodules develop rapidly into cavities (within days). The dynamic development helps differentiate it from malignancies. The cavities may show peripheral contrast enhancement. Wall thickness may vary considerably and is of no help in distinguishing from other entities. Pleural effusion is often present, which may develop into an empyema. Hilar and mediastinal lymphadenopathy may occur. Additional imaging findings include widening of the pulmonary artery due to increased pressure. If the thrombi reach the left heart, infarctions in the abdominal parenchymal organs, brain, and skin may occur (Figures 3, 4–6) [12,13].

### Mycobacterium Tuberculosis Infection

**Figure 7:**
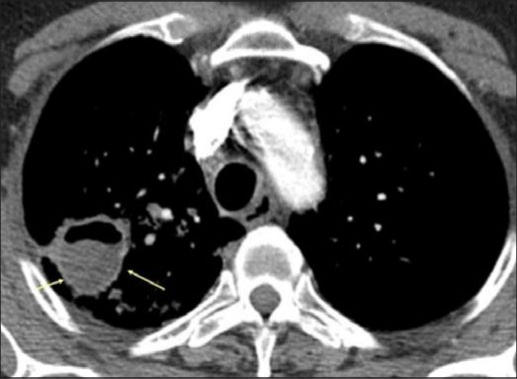
*Tuberculosis.* This 68-year-old male previously lived in areas of endemic tuberculosis. He was admitted with productive cough and fever in the evening in the past two weeks. Now he has hemoptysis with a C-reactive protein level of 120 mg/L. The initial radiograph showed a cavity. CT with contrast showed a fluid-filled cavity in the upper right lung, with faint contrast enhancement in the wall.

**Figure 8:**
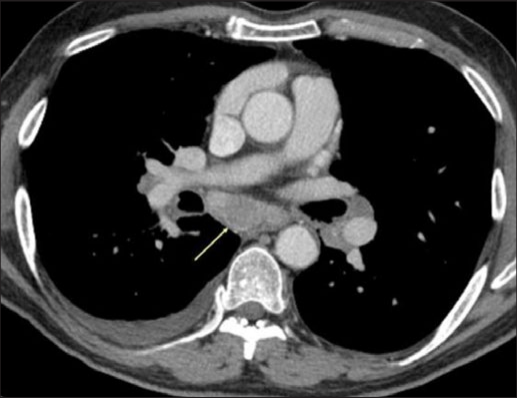
Rim enhancement is seen in the enlarged mediastinal lymph node.

**Figure 9:**
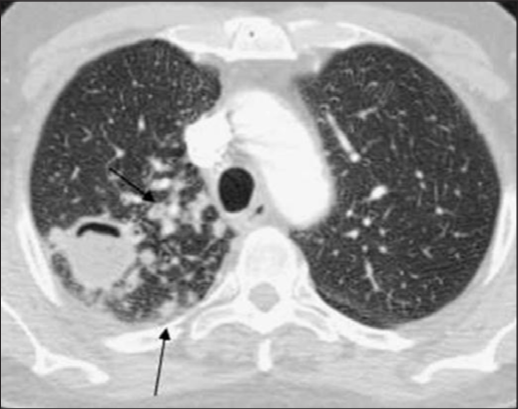
There are also multiple perilesional nodules (arrows).

**Figure 10:**
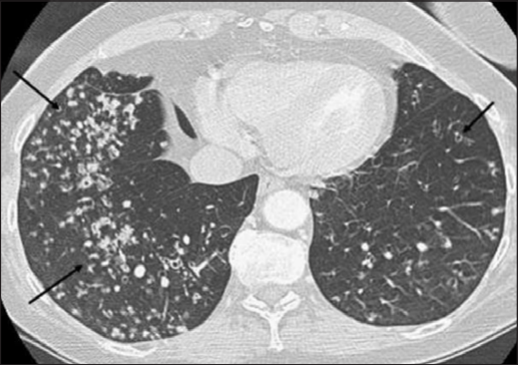
*Miliary tuberculosis.* A 65-year-old female with dyspnea was admitted as her pneumonia was not improving despite treatment. The CT was performed with a suspicion of pulmonary embolism. The images show multiple small cavitary nodules in the upper and lower lobes on both sides. Microbiology proved positive for tuberculosis.

Tuberculosis has an increasing prevalence after years of some decline. The clinical manifestation may be subtle or completely absent. Symptoms include low-grade fever, malaise, loss of weight, and, when the lungs are affected, cough, with or without hemoptysis [14,15]. The radiological division of primary and secondary features has been debunked [16]. However, a radiological pattern does exist; upper lobe cavitary disease is commonly seen in immunocompetent adults, while lower lung zone disease, adenopathy, and pleural effusions are commonly found in immunocompromised patients (children are considered in this group). The cavities in tuberculosis, which occur in 50 per cent of patients, are usually located in upper zones of the lobes. They are often surrounded by satellite nodules [15]. The cavity wall thickness may vary considerably, and the cavity wall may show rim enhancement on CT. If there is affection of the lymph nodes, one may see nodal rim enhancement around central necrosis (Figures 7, 8, 9). Pleural effusion may occur and is seen in 25 per cent of patients [15]. Miliary tuberculosis is hematogenous spread of disease, which presents as small, 2–3 mm sized nodules. They are usually located in the lower zones of the lobes and may cavitate (Figure 10) [15].

### Non-tuberculous Mycobacterial (NTMB) Infection

**Figure 11:**
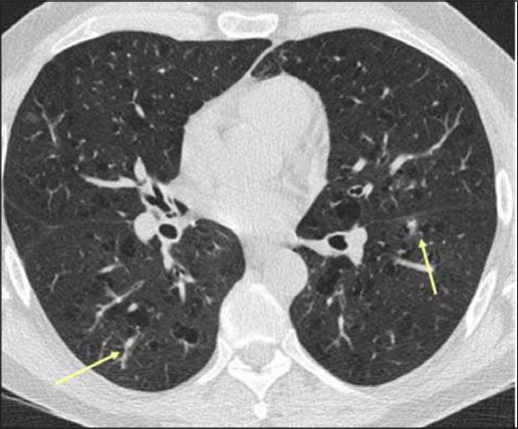
*Non-tuberculous mycobacterial infection.* A 50-year-old male and COPD patient had a routine HRCT exam that showed small nodules in both lungs (arrows).

**Figure 12:**
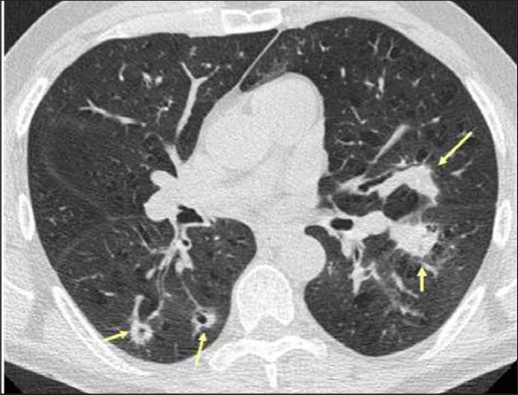
The patient returned with cough and malaise a year later. CT showed an increase in the size of the nodules as well as cavitation. Bronchial lavage produced acid-fast bacilli, later proved to be mycobacterium avium intracellulare.

Atypical mycobacterial or non-tuberculous mycobacterial (NTMB) infections are caused by mycobacteria other than *Mycobacterium tuberculosis*. They consist of dozens of different organisms, the most common are *M. avium-intracelluare* and *M. kansaii*. [17,18]. NTMBs are not as contagious as tuberculosis. Patients with preexisting pulmonary disease, such as chronic obstructive pulmonary disease, and the elderly are prone to NTMB infections. There are two main forms of presentation: classic and nonclassic. The classic form presents as cavitary disease in the upper zones of the lobes, with symptoms similar to tuberculosis but no hemoptysis. On imaging, there are nodules in all lobes, with a slight predilection for the apical and posterior segments. The nodules develop into cavities, as new nodules also occur. Wall thickness may vary from thin and smooth inner wall to thick and irregular inner wall. The cavitary nodules are seldom above 2.5 cm in size. Small calcifications may be seen. Mediastinal lymphadenopathy and pleural effusions are rare (Figures 11, 12). The nonclassic NTMB presents with chronic cough and as a bronchiectatic disease, with centrilobular nodules and tree-in-bud pattern in relation to the bronchiectasis. Cavitation and mediastinal lymphadenopathy are rare in nonclassic NTMB [18].

### Aspergillosis

**Figure 13:**
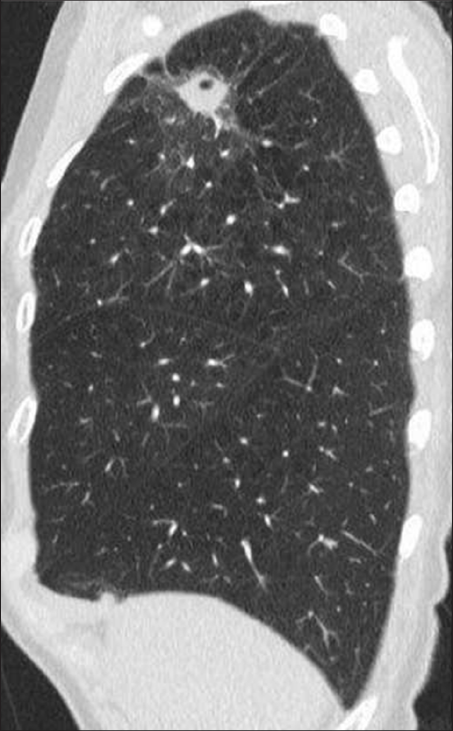
*Chronic necrotizing (semi-invasive) aspergillosis*. A 70-year-old female with long-standing asthma was treated with steroids. She was treated for pneumonia but still had rising CRP from 140 to 170mg/L. CT revealed a cavitary nodule in the right upper lobe with ground glass around it, a so-called halo sign (arrows).

**Figure 14:**
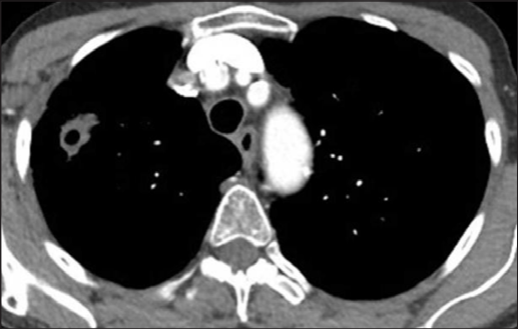
The nodule had a thick wall, but a smooth inner wall, and there were no lymph nodes in the mediastinum.

**Figure 15:**
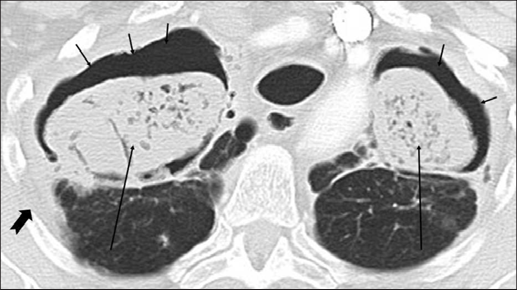
*Aspergilloma.* A 58-year-old male sarcoidosis patient also had known long-standing fibrosis. Routine CXR revealed opacities in the apical segments of both lungs. CT showed large content known fibrotic cysts apically with crescent-shaped air (short arrows) anteriorly due to large formed fungus balls (long arrows). There was local pleural thickening (thick arrow) (no symptoms).

Aspergillosis is caused by a fungus, *Aspergillus fumigatus*. Several forms of presentations exist: aspergilloma, allergic bronchopulmonary aspergillosis, chronic necrotizing (formerly semi-invasive) aspergillosis, and invasive aspergillosis. The two latter forms are seen in immunocompromised hosts, whereas aspergillomas are seen in patients with underlying cavities in the lungs. In invasive aspergillosis, the clinical presentation consists of prolonged symptoms of productive cough, fever, malaise, and sometimes hemoptysis [19,20]. On CT, initially there is consolidation, sometimes several, which may have a halo of ground glass surrounding it. The nodules may cavitate (Figures 13, 14) [19,20]. Aspergillomas are not true cavitary lesions but fungus balls that develop in patients with underlying diseases (tuberculosis, sarcoidosis) with preexisting cavities in the lungs. They may be completely asymptomatic, when they develop symptoms; the most common is hemoptysis due to affection of vessels [19,20]. On imaging, one may find a solitary cavitating or multiple cavitating opacities or masses with a crescent-shaped air collection in the nondependent part of the cavity. The crescent sign must be correlated to the clinical setting of underlying disease, as the sign is not unique for aspergilloma [20]. The mobility of the cavity’s contents may be used to differentiate it from other entities [19]. The wall of the preexisting cavity may be affected by the aspergilloma and become irregular, but wall thickness usually remains below 3 mm [20]. There may be local pleural thickening (Figure 15) [20].

## Systemic Diseases

### Granulomatosis with Polyangiitis

**Figures 16–17:**
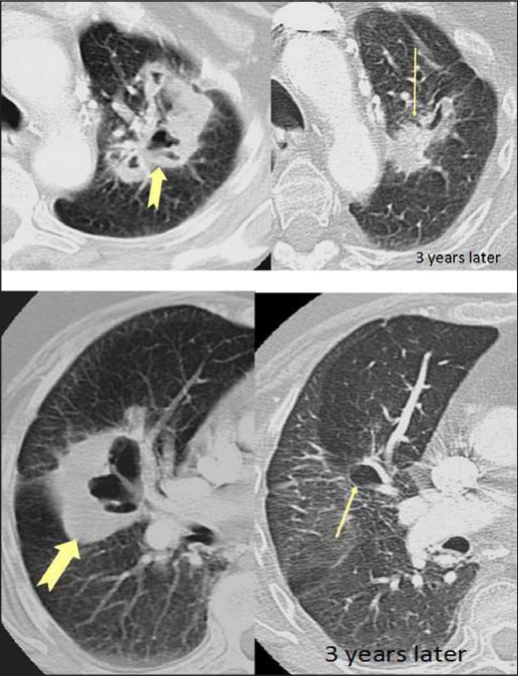
*Granulomatosis with polyangiitis.* A 74-year-old female was admitted due to dyspnea and productive cough, but no fever. The CT images show relatively large cavitary lesion in both lungs with thick, irregular walls (thick arrows). Three years later, the nodules had evolved into a cyst on the right side. On the left side, the nodule was smaller and no longer cavitary.

Granulomatosis with polyangiitis (GPA) is an autoimmune disease that causes vasculitis in the small vessels. Common organs affected are the upper and lower respiratory tracts and the kidneys. Nosebleeds and hemoptysis are the most common symptoms, and 95 per cent of all patients present with cough and dyspnea. Imaging reveals large pulmonary nodules and masses, usually 2–4 cm, and rarely up to 10 cm. Twenty-five per cent of all nodules cavitate. They are often they are located centrally but show no predilection for the upper or lower lungs. The wall thickness may vary considerably. The nodules sometimes have a halo surrounding them which is due to hemorrhage. About half of the nodules resolve over time in response to treatment; the remaining heal with residual fibrosis (or thin-walled cysts) or remain unchanged (Figures 16–17) [21,22]. GPA may also present as ground glass opacities or mosaic attenuation due to diffuse hemorrhage in the lungs.

### Rheumatic Nodules

**Figure 18:**
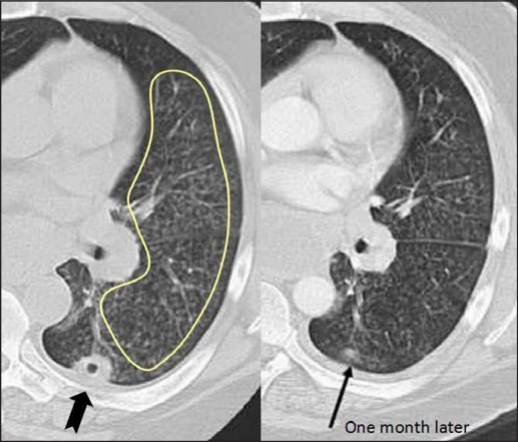
*Rheumatic nodule.* A 46-year-old male with long-standing rheumatoid arthritis was immunosuppressed due to ongoing methotrexate treatment. CT was requested for chronic cough. The CT showed multiple centrilobular nodules in the left lung (inside the marked area), which were bronchiolitis due to his RA. In addition, a subplueral cavitary nodule is seen (thick arrow). A month later, the nodule was slowly dissipating (arrow).

Cavitating nodular opacities in the course of rheumatic diseases are much rarer than interstitial pulmonary pneumonias and vasculitides. The nodules occur when epithelial cells cover a necrotic area, creating a necrobiotic nodule, which is the cause of the cavity. These are most often located in the periphery or subpleurally. They may vary in size and wall thickness. Normally, they are asymptomatic and resolve without specific treatment, but cough and hemoptysis have been reported in some cases (Figure 18) [23].

### Sarcoidosis

Sarcoidosis is a rare differential diagnosis of pulmonary cavitary nodules. Less than 1 per cent of patients with sarcoidosis develop cavitary nodules. They are reported as rounded or oval-shaped and are usually found in the perihilar or peripheral areas [24].

### Malignancies

**Figure 19:**
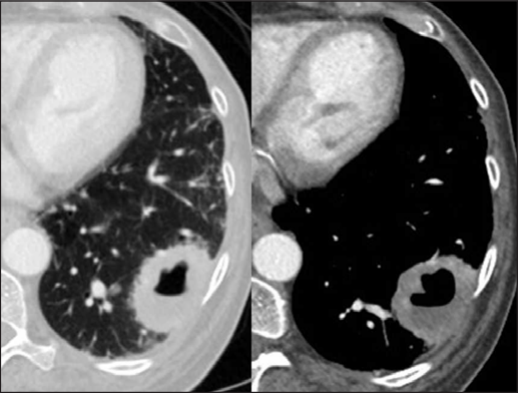
*Lung cancer.* A 65-year-old female patient with a smoking history of 50 years was admitted with hemoptysis and malaise, no fever. CT showed a large cavitary lesion with varying wall thickness. Note that without the clinical history this case is impossible to differentiate from the case presented in Figure 1.

**Figure 20:**
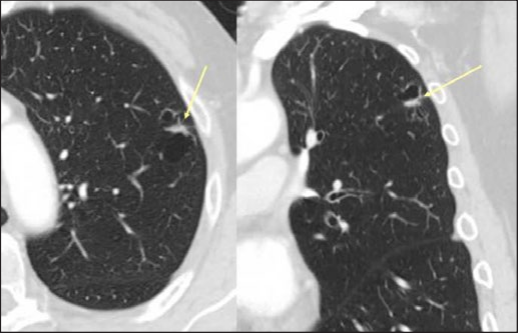
*Lung cancer.* A 70-year-old male patient was admitted due to dyspnea when supine, no fever or cough. In the left upper lobe, a mostly thin-walled multicystic lesion is seen, with a short thicker wall in-between (arrows). A biopsy proved adenocarcinoma.

The most commonly encountered solitary cavitary nodule in the lung is a malignant tumor [25]. They may occur anywhere in the lungs and have round or irregular shapes with a great variation in wall thickness. Wall thickness greater than 24 mm as well as perilesional consolidation may indicate malignancy, as mentioned earlier [18]. Of all bronchial carcinomas, 10–15 per cent are cavitary (Figures 19, 20).

**Figure 21:**
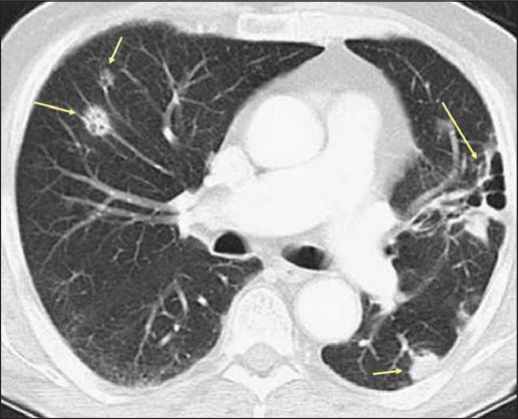
*Metastasis.* Pulmonary nodules with small and large cavitary components (arrows) are shown, which were metastases from an adenocarcinoma in the pancreas (not shown).

Pulmonary metastasis from squamous cell carcinomas, mainly from the gastrointestinal tract and breast, sarcomas, and adenocarcinomas frequently cavitate (Figure 21) [26]. On imaging, differentiating malignant tumors from other cavitary entities may be difficult, but the clinical history of weight loss and lack of acute symptoms such as fever may be helpful.

### Entities That Overlap with Cystic Diseases

**Figure 22:**
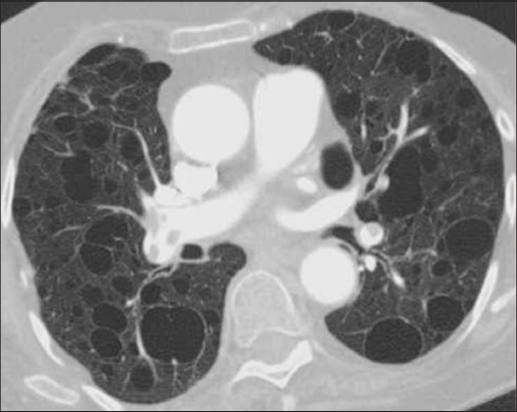
*LAM.* An 84-year-old female, previously operated on for an angiomyolipoma, was admitted with dyspnea. CT showed multiple thin-walled cysts with varying sizes.

**Figure 23:**
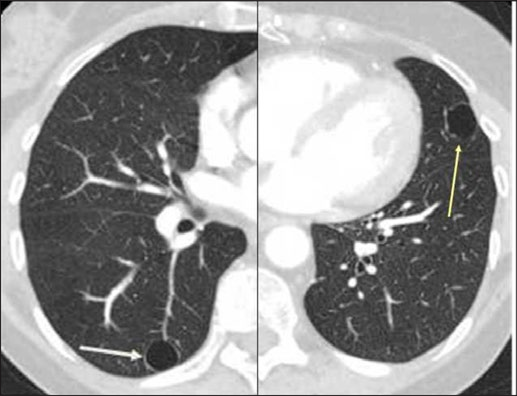
*LIP.* A 71-year-old female with systemic lupus erythematosus for decades was admitted with increasing dyspnea and chronic cough, prompting a CT request. Thin-walled cysts are seen on the right and left sides (arrows). Diagnosis was LIP, which is associated with SLE.

**Figure 24:**
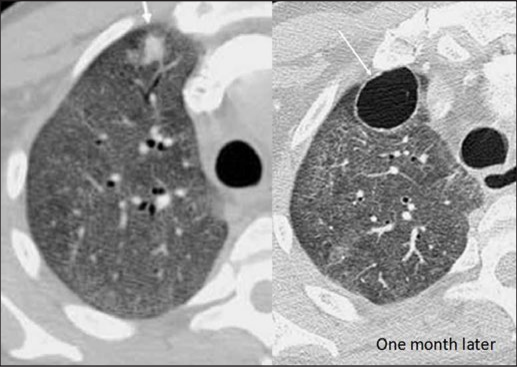
Pneumocystis jirovecii *infection.* A 40-year-old male was admitted with acute dyspnea. An initial CT was done for suspected PE. It showed extensive ground glass opacities in both lungs and a small consolidation in the right upper lobe. The patient was subsequently diagnosed with acquired immunodeficiency. One month later (HRCT) the ground glass is less extensive, and the nodule has developed into a thin-walled cyst.

**Figures 25–26:**
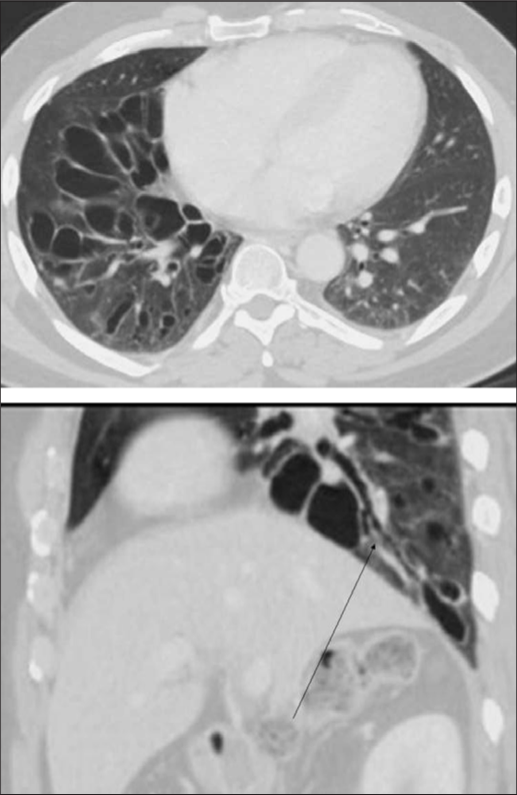
*Cystic bronchiectases*. A 51-year-old male was admitted with abdominal pain. An incidental finding in the basal lung right side showed cysts (grape-like clusters) with direct connection to the bronchial system (long arrow). The severe but unilateral affection excluded a systemic disease and was consistent with cystic bronchiectases, probably secondary to an infection.

Langerhans cell histiocytosis (LCH), lymphangioleiomyomatosis (LAM), lymphocytic interstitial pneumonia (LIP), and findings of pneumocystis jirovecii infection are all cystic lung diseases (Figures 22, 23, 24, 25–26). Cystic bronchiectasis may also be a differential diagnosis. However, wall thickness as well as clinical findings are important criteria to differentiate between cavitary and cystic lung diseases [27,28,29,30,31].

## Conclusion

To summarize, cavitary pulmonary lesions are caused by a number of varying disease entities. Paying close attention to disease-specific CT findings combined with the clinical history and findings are important tools leading to the correct diagnosis.
